# Prolene Suture Suspension Within a Multimodal Facial Rejuvenation Protocol: A 30-Year Experience

**DOI:** 10.1007/s00266-026-06129-y

**Published:** 2026-07-07

**Authors:** Onur Erol, Serhat Şibar, Sadri Ozan Sözer

**Affiliations:** 1 ONEP Plastic Surgery Science Institute, Istanbul, Turkey; 2 Department of Plastic and Reconstructive Surgery, Department of Plastic Reconstructive and Aesthetic Surgery, Gazi University Hospital, 14th floor, 06500 Ankara, Turkey; 3 Private Practice, El Paso Cosmetic Surgery, El Paso, Texas USA

**Keywords:** Minimal invasive surgical procedures, Rejuvenation, Sutures

## Abstract

**Background:**

With rising life expectancy and increasing emphasis on quality of life, demand for facial rejuvenation procedures has markedly grown. This study describes a 30-year single-surgeon experience with a minimally invasive suture suspension technique and reports descriptive findings on patient satisfaction in a retrospective case series.

**Methods:**

A retrospective chart review was conducted on 4400 patients (3800 female and 600 male) aged 18 to 65, who underwent brow, temporal, or cheek suspension. All procedures were performed without surgical dissection, using small stab incisions for permanent suture placement. In selected cases, the technique was combined with micro-fat grafting or laser resurfacing. Patient satisfaction was assessed during routine follow-up visits using a non-validated 5-point Likert scale as a descriptive measure.

**Results:**

A total of 2500 brow and 1900 cheek/temporal suspensions were performed. Among patients with documented ratings, 93.2% reported satisfaction scores of 4 or 5 on this descriptive scale. Common early postoperative findings included transient swelling, dimpling, and bruising. Revision under local anesthesia was necessary in 2.3% of cases. The longevity of results was estimated from photographic documentation and patient feedback in a subset of patients. In this multimodal treatment cohort, aesthetic improvements appeared to be maintained over time in selected patients. However, the duration of effect could not be systematically quantified because follow-up was retrospective and not standardized.

**Conclusions:**

Within the limitations of this retrospective descriptive case series, this suture suspension technique appears to be a safe, well-tolerated, and minimally invasive option for selected patients with early facial aging, and it can be combined with adjunctive cosmetic procedures as part of a multimodal protocol.

**Level of Evidence IV:**

Facial Surgery. “This journal requires that authors assign a level of evidence to each article. For a full description of these Evidence-Based Medicine ratings, please refer to the Table of Contents or the online Instructions to Authors www.springer.com/00266.”

**Supplementary Information:**

The online version contains supplementary material available at https://doi.org/10.1007/s00266-026-06129-y.

## Introduction

With increasing life expectancy and greater emphasis on aesthetic well-being, the demand for facial rejuvenation has risen substantially. Data from the American Society of Plastic Surgeons and the American Society for Aesthetic Plastic Surgery show marked growth in noninvasive treatments such as botulinum toxin injections, whereas surgical facelift volumes have increased more modestly [1]. Although these reports are dated, they illustrate a persistent shift in patient preferences toward less invasive procedures, a trend that continues to shape current aesthetic practice. Many patients are reluctant to undergo extensive facial surgery because the face plays a central role in self-perception and social interaction. Minimally invasive facial rejuvenation techniques are attractive in this group because they offer lower morbidity, shorter recovery, and a favorable safety profile [2, 3]. Brow ptosis, which contributes to a tired appearance and upper eyelid fullness, is a common concern. Yet, even endoscopic brow lifting may be declined by patients who seek milder interventions.

The technique evaluated in this study is an evolution of the brow suspension method first introduced by the senior author in 1996 and published in 2002 [4]. It uses permanent sutures passed through small stab incisions to correct brow ptosis without wide dissection.

The current study extends this original concept by documenting its application to broader facial regions and by descriptively summarizing three decades of single-surgeon clinical experience, including complications and patient-reported satisfaction. In doing so, it also highlights the limitations of commercial thread lifting systems that became popular in the early 2000s, many of which provided only short-lived improvement, typically 7–8 months, and inconsistent long-term results. In contrast, the present technique employs permanent sutures anchored beneath the galea to apply a vertical vector to the superficial fascia and platysma, making it anatomically and conceptually distinct from conventional thread lifts. Over time, it has been refined with custom-designed instruments that enable minimally invasive brow and midface suspension without surgical undermining. The present report summarizes clinical outcomes and complication rates in a large single-surgeon cohort of 4400 patients treated with this standardized protocol. This work is therefore presented as a large, historical retrospective description of a single-surgeon clinical experience over three decades, rather than a formal outcomes-based analysis.

## Methods

Over the past 30 years, 2500 brow and 1900 cheek and temporal suspensions were performed on 4400 patients (3800 female and 600 male) aged 18 to 65.

The inclusion criteria were patients with mild to moderate facial aging, evident brow ptosis or midface descent, and refusal or ineligibility for conventional surgical rejuvenation. The exclusion criteria were patients with severe facial ptosis, prior facial surgery in the treatment area, or connective tissue disorders.

Patient satisfaction was assessed during routine follow-up visits using a 5-point Likert scale ranging from 1 (very dissatisfied) to 5 (very satisfied), typically within 1 to 12 months post-procedure, depending on patient availability. This pragmatic clinical tool was used to capture patients’ global impressions of improvement in everyday practice and was not formally psychometrically validated. This measure should therefore be considered a descriptive indicator of patient perception rather than a validated outcome assessment tool. Follow-up visits were generally scheduled during the early postoperative period, with additional visits as needed. Pre- and postoperative photographs were reviewed to assess aesthetic outcomes. These evaluations were performed by the senior author based on available standardized angles. While standardized photographic documentation and survey timing varied, especially in earlier years, all available records were categorized and analyzed accordingly. Complications were recorded based on available clinical records during routine follow-up visits. Given the retrospective design and long study period, systematic prospective complication tracking was not uniformly applied, particularly in earlier years; however, all clinically significant complications requiring intervention were documented.

This approach was offered to patients who either declined or were not optimal candidates for surgical face and neck lifting. In many cases (72.7%), the suture suspension procedure was performed as part of a multimodal rejuvenation protocol and combined with micro-fat grafting and/or fractional CO₂ laser resurfacing (Table 1). In the present study, clinical outcomes and complications are reported for the entire treatment cohort and should therefore be interpreted as reflecting this protocol rather than the suture technique in isolation. Accordingly, the effects of suture suspension cannot be separated from those of adjunctive procedures within this treatment framework In combined cases, Fraxel Repair fractional CO₂ laser resurfacing was performed to improve skin texture and elasticity. Autologous micro-fat grafting was performed to restore facial volume where indicated. Fat was harvested from the abdominal or thigh region, processed by decantation, and injected into the perioral, chin, and malar areas in amounts ranging from 10 to 30 cc, depending on the patient’s needs.

**Table 1:** Type of adjunctive procedures of the study cohort

Type of procedure	Number of patients (n)
Laser resurfacing	274 (6.2%)
Micro-fat grafting	2261 (51.4%)
Combination of the above	665 (15.1%)
Total	3200 (72.4%)

## Operative Technique

### Eyebrow Suspension

Preoperative marking was performed with the patient in the supine position to capture the desired lifted brow contour. Four stab incision sites were identified: two approximately 1–2 cm above the medial and lateral brows, and two within the temporal hairline aligned with the tail of the brow. Following local anesthesia with 1% lidocaine containing 1:100,000 epinephrine, small stab incisions were created using a #11 scalpel blade. A custom-designed hook needle was used to pass a 4–0 prolene suture through the subdermal plane, forming a supportive loop from medial to lateral. A straight hook was then used to tunnel the suture between the lateral brow incision and the temporal hairline incision. Both suture limbs were directed toward and exteriorized from the medial temporal incision.

**Fig. 1:**
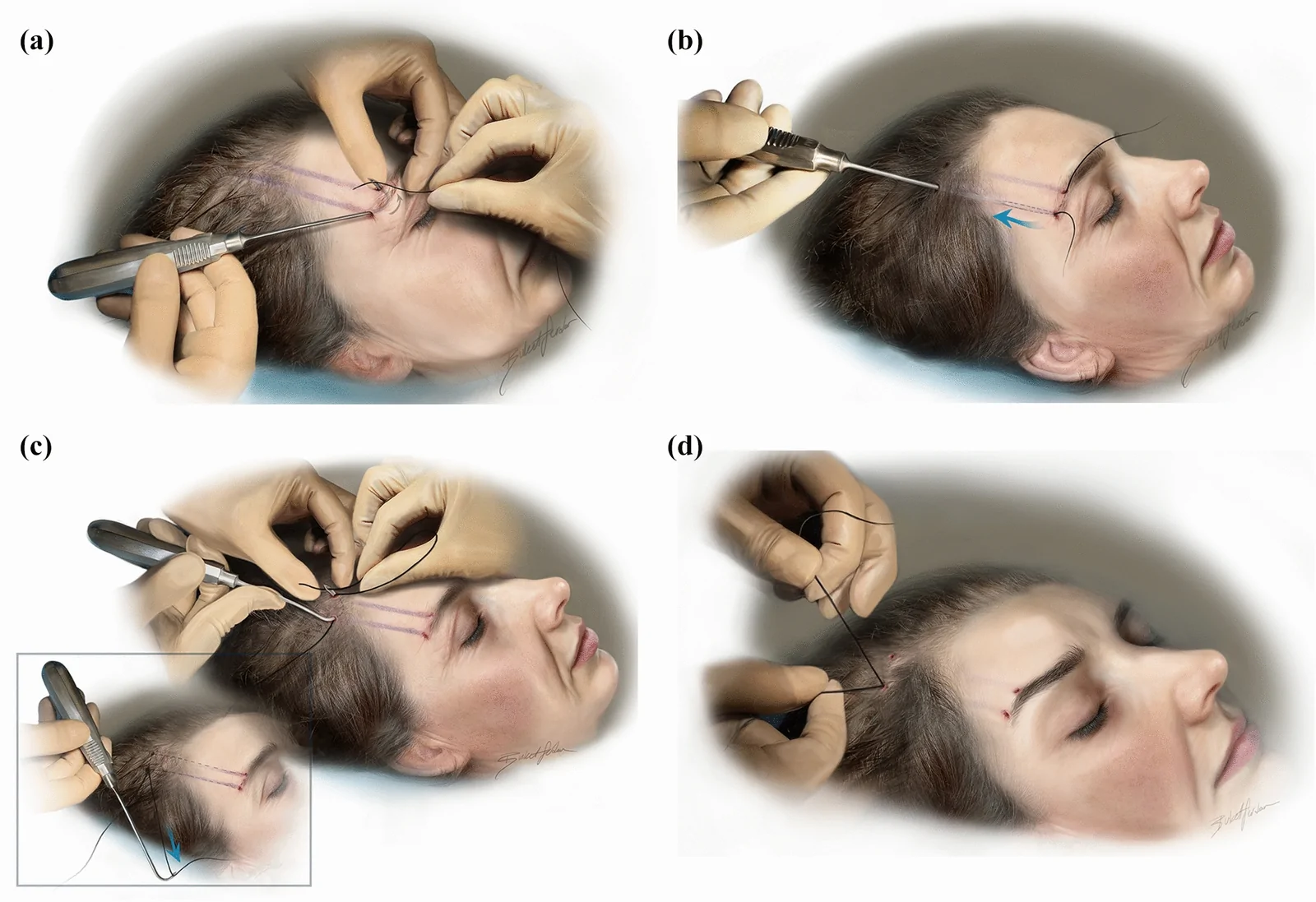
Passing the prolene suture with a curved hook through stab incisions in the eyebrow (**a**) Passing the suture to the temporal region with a straight hook (**b**) Moving suture ends to the same temporal incision with a curved hook (**c**) Elevation is achieved in the eyebrow by tying the suture ends (**d**)

The suture was tied under moderate tension, anchoring it to the deep dermis and superficial fibrofatty connective tissue, thereby maintaining the elevated brow position visualized during supine marking. Each suspension loop was secured with six square knots to prevent slippage associated with the memory of Prolene material. Additional loops could be placed as needed. This procedure could be performed as a standalone technique or combined with upper eyelid blepharoplasty using the same incisions (Fig. 1).

### Temporal Suspension

**Fig. 2:**
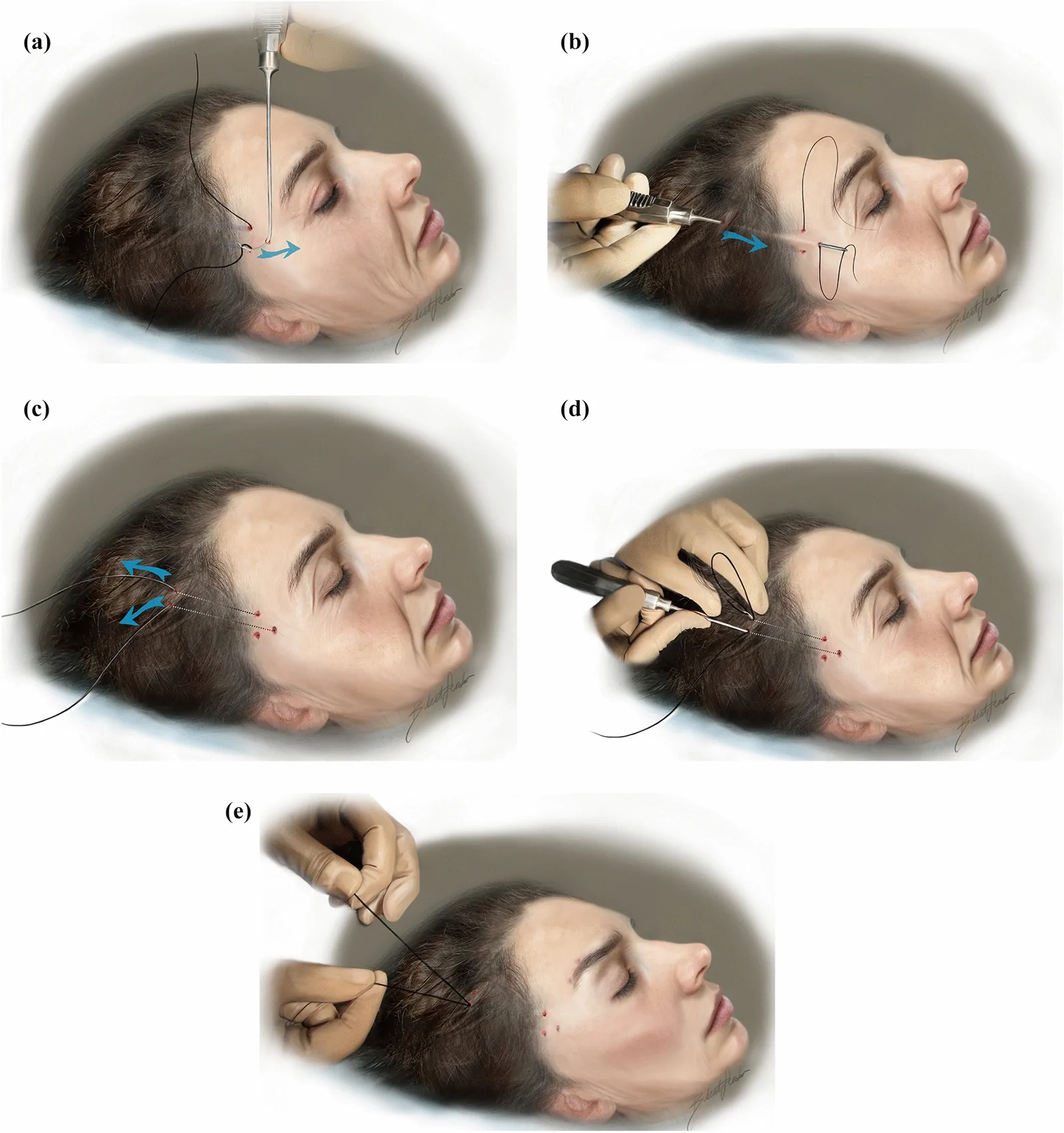
Prolene suture was introduced through three stab incisions made adjacent to the temporal hairline for temporal suspension (**a**) Passing the suture to the temporal region with a straight hook (**b**) Passing the other end of the suture through the second temporal stab incision (**c**) Moving suture ends to the same temporal incision with a curved hook (**d**) Elevation is achieved in the temporal region by tying the suture ends (**e**)

Temporal suspension involved five stab incisions: three placed along the temporal hairline (anterior, middle, and posterior) and two within the superior hairy temporal scalp. These incision points corresponded to the desired lifting vector and anchorage site. Using a curved hook, a 2–0 prolene suture was introduced through the subdermal and superficial fascial plane to engage the temporal SMAS and fibro-adipose tissue. The lateral limb was passed with a straight hook toward the posterior scalp incision, while the medial limb was tunneled using a curved hook. Both limbs were exteriorized from the same posterior scalp incision. The knot was securely tied and buried in the deep temporal fascia. This approach provided a lateral and slightly superior vector to support the lateral brow and temporal soft tissue (Fig. 2)

### Cheek Suspension

**Fig. 3:**
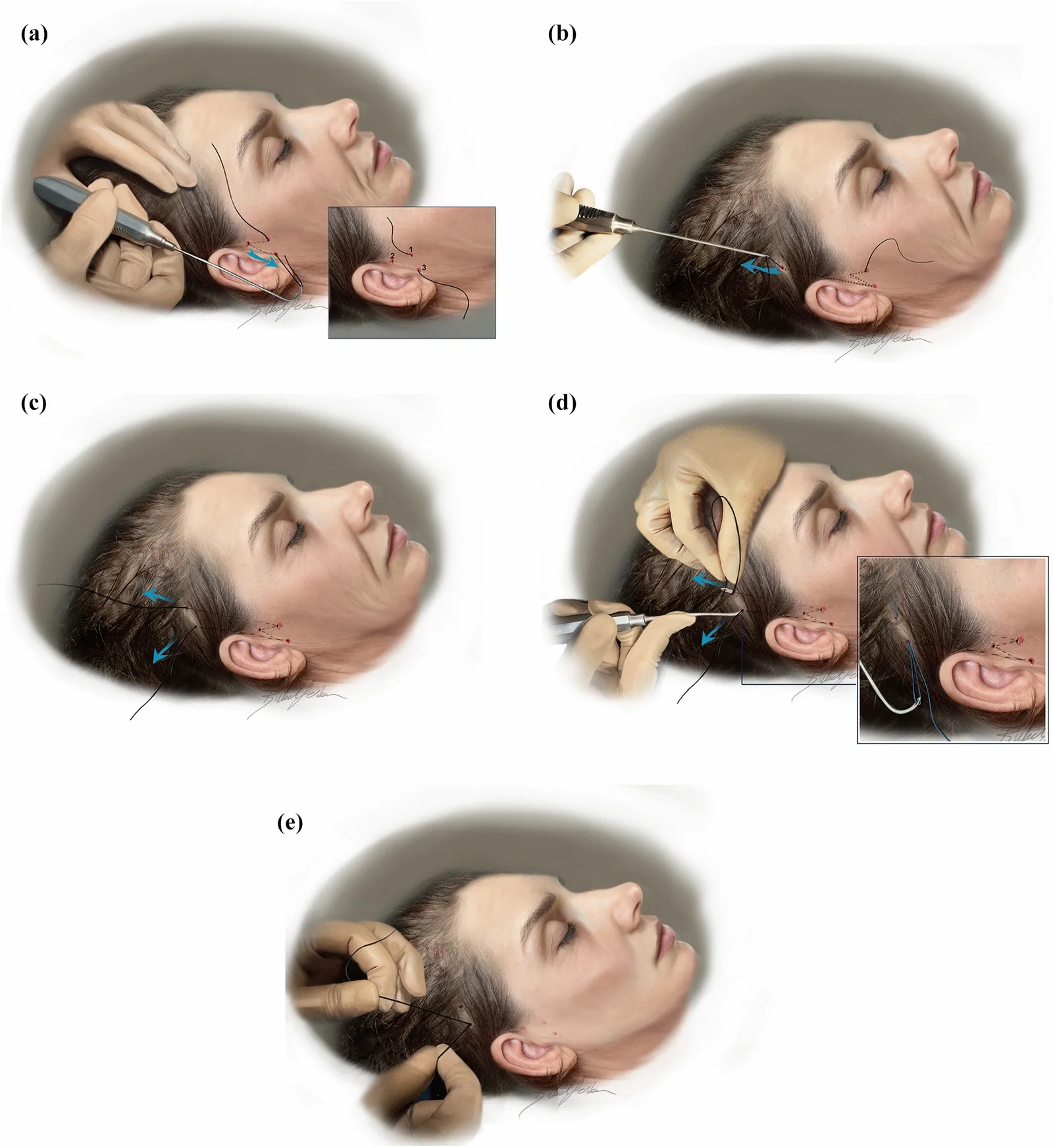
Creating a loop in the preauricular region with three stab incisions for cheek suspension (**a**) Passing the loop created in the preauricular region to the temporal scalp (**b**) Passing the suture to the temporal region with a straight hook (**c**) Moving suture ends to the same temporal incision with a curved hook (**d**) Elevation is achieved in the cheek region by tying the suture ends (**e**)

Cheek suspension required five small incisions: three just anterior to the sideburn and two in the temporal scalp region. The markings were based on individual ptosis patterns and intended lift trajectories. A loop was formed using a 2–0 prolene suture that passed through the subcutaneous and deep tissue layers, engaging both the dermis and the superficial musculoaponeurotic system overlying the malar region. The lateral limb was passed posteriorly toward the temporal incision using a straight hook, and the medial limb was retrieved using a curved hook beneath the galea. The final knot was tied under adequate tension and buried within the deep temporal fascia, effectively lifting the midface, jawline, and preauricular tissues in a vertical or oblique vector (Fig. 3).

In selected patients with fuller or heavier midface or jowl regions, pre-procedural contouring with micro-liposculpture improved suspension outcomes. This was performed using a 1.5–2.0 mm blunt-tip microcannula inserted through a small stab incision, typically near the sideburn or within the temporal hairline. The cannula was advanced in a superficial subcutaneous plane under local anesthesia, and gentle aspiration was used to debulk excess fat in the malar or jowl areas. This maneuver reduced mechanical resistance and enhanced the effectiveness of the upward lifting vector.

Across all procedures, there was no wide undermining or skin flap elevation. Suspension was achieved purely through deep-plane suture engagement and tension-based fixation. The use of five stab incisions and deep suture placement without dissection justifies the designation “minimally invasive facial suspension.” A demonstration of the temporal suspension procedure is provided as Supplemental Digital Content-1.

## Results

A total of 4400 patients underwent treatment. The age distribution was as follows: 2835 patients (64.4%) were between 18 and 40 years, 1145 patients (26.0%) between 40 and 50 years, and 420 patients (9.5%) between 50 and 65 years. The majority of patients benefited from this approach and reported high satisfaction, particularly after reviewing their before-and-after photographs. According to the non-validated 5-point Likert scale, 93.2% of patients with recorded scores rated their satisfaction as 4 (satisfied) or 5 (very satisfied) (Table 2). These findings should be interpreted with caution given the non-validated nature of the assessment tool and the absence of standardized outcome measures.

**Table 2:** Patient distribution according to Likert scale scores

Likert scale score	Number of patients (n)
1 (Very dissatisfied)	17 (0.4%)
2 (Dissatisfied)	76 (1.7%)
3 (Neutral)	208 (4.7%)
4 (Satisfied)	3498 (79.5%)
5 (Very satisfied)	601 (13.7%)

### Early Postoperative Findings

Patients commonly described a fresher, more rested appearance, especially in the periorbital and midface regions. It is important to inform patients that this is a suspension-based procedure and should not be expected to replicate the results of a full face or neck lift. The aesthetic improvements are more subtle and best suited for those with early signs of facial aging. The most frequently observed early postoperative findings were swelling, bruising, and dimpling (n=1179; 26.7%), all of which resolved with conservative management.

### Complications

Asymmetry was noted in 67 cases (1.5%), with revision under local anesthesia required in 28 of these patients (<1%). Suture-related issues—such as subcutaneous visibility, suture rupture, or suture exposure—occurred in 65 patients (1.5%). These included 26 cases in the brow group and 39 in the temporal and cheek group. All suture-related problems were managed with minor revision under local anesthesia. Infection occurred in 13 cases (0.3%), and in nine of these, sutures were removed due to poor response to antibiotics. Once signs of infection had resolved, repeat procedures were performed electively. Notably, no cases of allergic reaction to suture material, skin necrosis, or facial nerve injury were observed. Likewise, we did not encounter any intraoperative or postoperative instances of tissue tearing attributable to Prolene suture traction (i.e., cheesewiring). Careful suture placement and controlled tension likely prevented focal overload, thereby preserving soft tissue integrity. Although 65 patients experienced minor suture-related events—such as subcutaneous visibility, rupture, or exposure—none involved mechanical laceration of the surrounding soft tissue.

Taken together with the 28 cases of early asymmetry requiring revision, these accounted for the majority of secondary procedures. In total, 102 patients (2.3%) underwent minor revisions under local anesthesia, as summarized in Table 3. In this study, revision procedures were defined as any secondary intervention performed under local anesthesia to address asymmetry, suture-related issues, or patient dissatisfaction.

**Table 3:** Types of complications encountered in the study cohort

Type of complication	Number of patients (n)
Early asymmetry	67 (1.5%)^a^
Suture-related problems (visibility, rupture, exposure)	65 (1.5%)^b^
Infection	13 (0.3%)^c^
Revisions under local anesthesia	102 (2.3%)
Facial nerve injury / necrosis	0 (%0)

a: Revision was performed in 28 of 67 cases.

b: All suture-related problems were revised under local anesthesia.

c: Revision was performed in 9 of 13 cases.

Because multimodal combinations were used in most patients and adjunctive procedures were not standardized over the 30-year study period, we did not perform a separate statistical analysis restricted to the pure suture subgroup, as this would have introduced substantial selection bias. Consequently, overall aesthetic outcomes in this series cannot be attributed solely to the suture suspension component.

### Brow Suspension

This approach yielded significant improvements in periorbital aesthetics. When combined with volumetric contouring, laser resurfacing, or upper blepharoplasty, the rejuvenation effect was further enhanced. No cases of forehead sensory change were reported. Slight dimpling occasionally occurred during the early postoperative period but resolved spontaneously as edema subsided.

### Temporal Suspension

Temporal suspension provided noticeable elevation in the malar and zygomatic regions. The procedure produced a vertical lifting effect by engaging both skin and platysma in the subdermal plane. Patients often reported a sensation of tightness during local anesthesia. Results were more pronounced in patients with thinner facial soft tissue. For individuals with fuller or heavier faces, pre-procedural contouring using micro-liposculpture can improve suspension outcomes. Transient dimpling of the temporal region is resolved within four to six weeks.

### Cheek Suspension

This method enhanced the lower cheek and jowl area, particularly in the preauricular zone. In cases with localized jowl fullness, micro-liposuction was performed prior to suture suspension. As with midface suspension, the vertical lifting vector targets both the skin and the underlying superficial fascia. Patients often reported subjective tightness in the cheek region during the procedure. Early dimpling near the sideburn typically resolved within four to six weeks.

### Longevity of Lifting

Longevity was estimated based on available follow-up photographs, physical examination findings during routine clinical visits, and subjective patient feedback regarding the persistence of aesthetic improvements. Because follow-up visits were not scheduled at predefined intervals and no survival- or time-to-event analyses were performed, this section provides a qualitative overview of perceived durability rather than a precise estimate of time to recurrence.

### Longevity of Brow Suspension

**Fig. 4:**
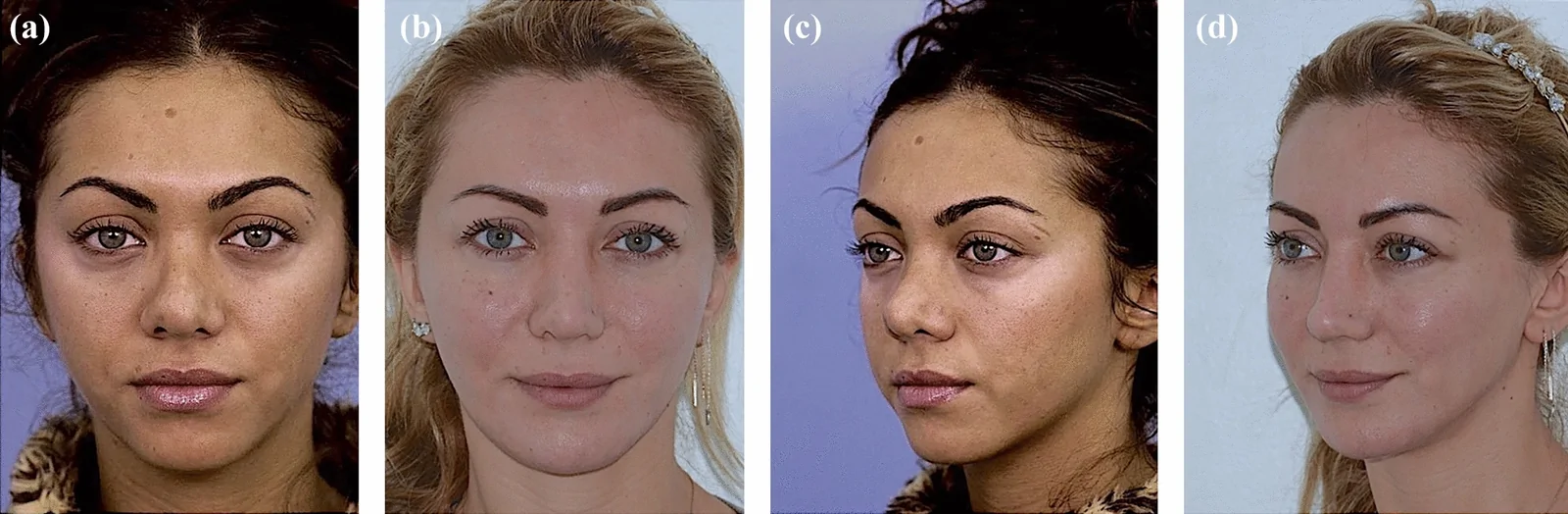
A 26-year-old woman who underwent brow and temporal suture suspension, facial micro-fat injection, and secondary rhinoplasty. Preoperative (**a**, **c**) and postoperative 14th-year appearance (**b**, **d**). Representative long-term example; not reflective of standardized cohort-wide follow-up.

**Fig. 5:**
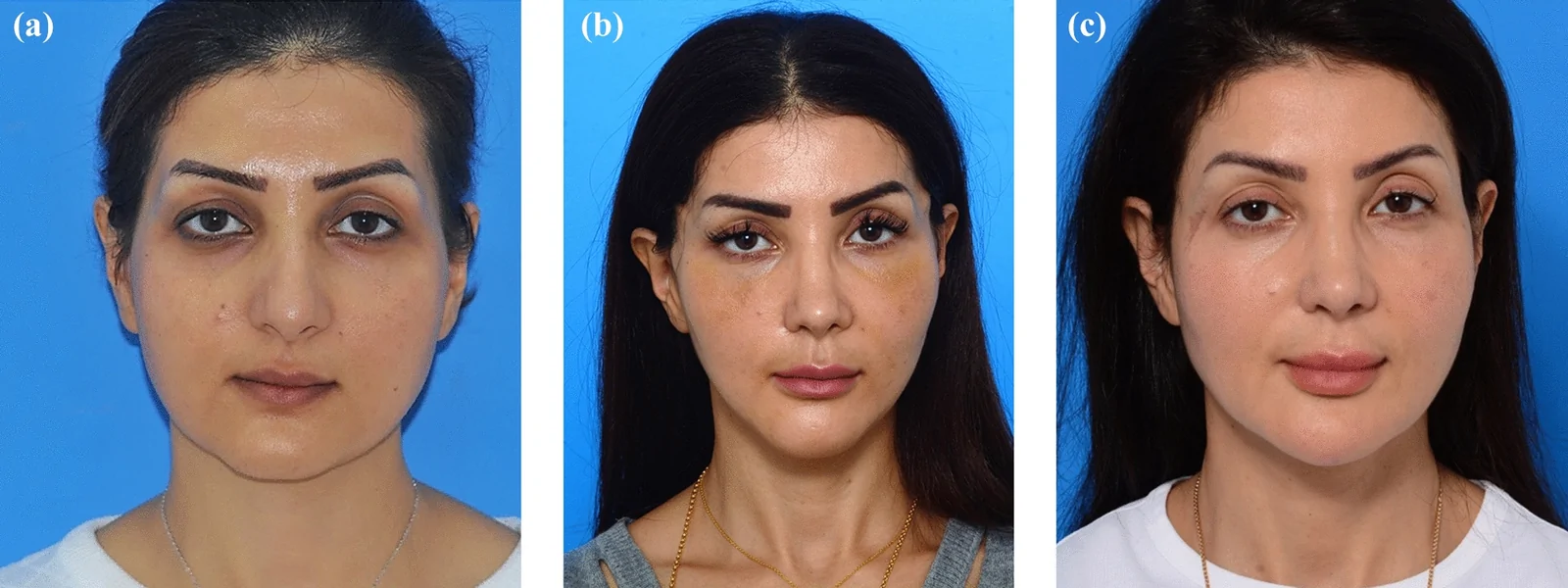
A 32-year-old woman who underwent brow and temporal suture suspension, rhinoplasty, and facial micro-fat injection. Preoperative (**a**) and postoperative 5th (**b**) and 11th year (**c**) appearance. Representative long-term example; not reflective of standardized cohort-wide follow-up.

When the patient is supine, the brow is naturally elevated; when pushed down, it partially covers the upper eyelid. After suture suspension, the new brow position is fixed and cannot be pushed down. In our practice, we have observed a few anecdotal cases, including the senior author, in whom brow elevation has appeared to remain stable over extended follow-up in selected anecdotal cases after suspension; however, these outliers should be interpreted cautiously and cannot be extrapolated to the overall cohort. Patients remain happy and often request additional facial suspensions or micro-fat injections for further rejuvenation (Figs 4 and 5).

### Longevity of Temporal and Cheek Suspension

**Fig. 6:**
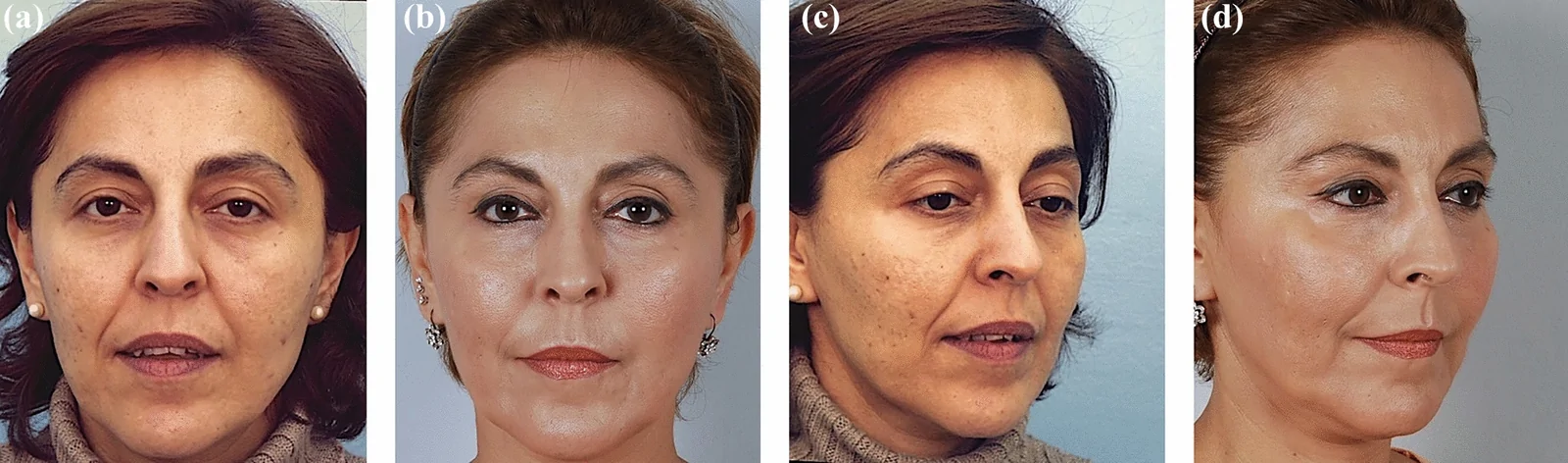
A 47-year-old woman who underwent brow and cheek suture suspension and facial micro-fat injection. Preoperative (**a**, **c**) and postoperative 6th year appearance (**b**, **d**).

The duration of the midface and cheek-lifting effects varied among individuals. Factors such as age, skin elasticity, facial volume, smoking status, and weight fluctuations appeared to influence the longevity of results. In patients with longer-term clinical or photographic follow-up, aesthetic improvement in the midface region often appeared to be maintained for several years; however, because follow-up intervals and documentation were variable, the exact duration of effect could not be determined, and repeat minimally invasive procedures were occasionally performed to maintain the rejuvenated appearance (Fig. 6).

## Discussion

People often feel apprehensive about surgery, particularly when it involves the face. Many therefore prefer noninvasive or minimally invasive options for facial rejuvenation. In this context, techniques that reduce operative trauma and recovery time while still providing visible improvement are of particular interest. Although several effective surgical procedures for forehead rejuvenation have been described, the number of patients who choose these operations remains limited [5–27]. Less invasive approaches, such as limited-incision foreheadplasty and endoscopic forehead lifting, have increased interest in brow rejuvenation [5, 28]. Suture suspension has also been incorporated into facial aesthetic surgery, including malar fat pad elevation in midface rejuvenation, canthopexy, and midface suspension to soften deep nasolabial folds [29–34]. As with any minimally invasive method, success depends on careful patient selection. Patients with mild soft tissue descent and relatively fine wrinkles are the best candidates for suture suspension, whereas heavy faces with marked ptosis are more likely to have suboptimal outcomes. [35, 36]

The present report is not designed as a formal outcomes study but rather as a large retrospective descriptive case series. Over three decades, documentation practices evolved and objective outcome instruments, blinded assessments, and comparison groups were not applied in a systematic fashion. As such, the data should be interpreted as a detailed account of clinical experience, complication patterns, and patient-reported satisfaction within a multimodal, minimally invasive rejuvenation protocol, rather than as definitive evidence of efficacy. Accordingly, objective anthropometric measurements, blinded photographic assessments, and validated patient-reported outcome instruments were not systematically applied in this series.

The suspension technique evaluated in this series was developed by the senior author to elevate the brow and midface without wide surgical dissection. The core principle is to counteract gravity by fixing the tissues in a position that resembles their appearance when the patient is lying supine, when the face often looks fresher and less tired.

Brow suspension using only sutures has been particularly useful for patients who are not interested in formal surgical brow lifts. However, the technique is not limited to this group. It can be safely combined with upper lid blepharoplasty, micro-fat grafting, and fractional laser resurfacing to enhance overall aesthetic outcomes. In the literature, combining suture suspension with other treatment modalities has been shown to improve the quality and longevity of results [37]. In selected patients with midface or jowl volume excess, micro-liposuction reduces bulk and tissue resistance, whereas collagen contraction induced by radiofrequency may facilitate soft tissue repositioning. Together, these adjunctive procedures can mitigate some limitations of each individual technique and provide a synergistic effect [37, 38]. Ogilvie et al. reported that the benefits of a such combined approach can be maintained for up to 36 months [39]. In our experience, many patients treated with this multimodal minimally invasive protocol have maintained visible improvement in brow and midface position for several years. Importantly, these impressions of longevity are derived from selected long-term observations and subjective clinical experience, rather than structured survival analysis or standardized follow-up intervals. For this reason, the present study does not attempt to isolate the independent effect of the suture suspension technique, but rather reflects outcomes observed within a combined, real-world clinical protocol. However, because adjunctive procedures such as micro-fat grafting, micro-liposuction, and laser resurfacing were frequently performed and follow-up was not standardized, longer-lasting results should be regarded as anecdotal rather than generalizable and cannot be attributed to the suture suspension component alone. The permanent sutures, buried deep under the galea, have not caused discomfort in our long-term follow-up. An important technical concern with suture-based lifting procedures is “cheesewiring,” in which the suture gradually cuts through soft tissues and the lifting effect diminishes over time. In the present technique, this risk is reduced by using a favorable anatomic plane for suspension. The suture passes through the subdermal and superficial fascial layers, including fibro-adipose tissue and galea, which provide broader resistance and more even tension distribution. Anchoring is performed without excessive traction or point fixation, allowing progressive tissue adaptation and minimizing tissue tearing or suture migration.

From a biomechanical standpoint, nonabsorbable Prolene sutures anchored in galeal and fascial planes have the potential to provide more durable structural support than absorbable barbed threads and many commercial lifting systems. Absorbable systems composed of PDO or PLA often show limited structural persistence, with clinical effects typically lasting 6–12 months [2, 40–42]. By contrast, Prolene provides long-lasting tensile strength, low tissue reactivity, and stable handling characteristics [34, 43, 44]. However, the present retrospective case series was not designed to compare the clinical longevity of different materials directly, and our data do not allow definitive conclusions regarding the independent long-term efficacy of Prolene suspension. These properties, together with its wide availability and relatively low cost, make Prolene an attractive option for minimally invasive facial suspension. In our experience, patients aged approximately 30–45 years with good skin tone benefit most from this technique. This age distribution likely contributed to the favorable outcomes observed, as patients with early stage facial aging are generally more suitable candidates for minimally invasive suspension techniques. In patients with fuller faces, micro-liposuction is often performed before suspension, whereas in individuals with marked laxity and advanced aging, a formal facelift remains the more appropriate option.

The anatomy of the lateral brow differs from that of the medial brow, as demonstrated by Knize [30]. The lateral brow has fewer robust connections to deep tissues and relatively looser skin. For this reason, many forehead rejuvenation techniques do not consistently achieve satisfactory lateral brow elevation in patients with marked laxity in this region. In such cases, passing a suture bite through the fibro-adipose tissue of the lateral brow and anchoring it to the temporal fascia can enhance lateral elevation during either endoscopic or non-endoscopic forehead lifting. When upper blepharoplasty is planned, the lateral portion of the blepharoplasty incision can be used instead of lower stab incisions, and the results can be complemented with a modest brow elevation using the same suspension principle. Similarly, the brow suspension technique can help correct certain asymmetries that persist after surgical brow lifts, particularly in patients with residual lateral laxity.

Over the past decades, numerous methods for brow and midface rejuvenation have been proposed, ranging from direct brow excision to pretrichial, bicoronal, and endoscopic lifts, as well as various implant- and thread-based systems. None has emerged as a universal gold standard, reflecting ongoing debate about the ideal balance between invasiveness, durability, and risk. The present suture suspension technique should therefore be regarded as one additional option within this spectrum, best suited for patients who desire a minimally invasive approach and accept the possibility of more modest change than with formal lifting procedures. In daily practice, we have also observed that individual muscular activity influences the degree and persistence of elevation, particularly in patients with strong brow depressor function or pronounced midface animation. Although quantitative data on this factor were not collected in the current series, preoperative evaluation of dynamic facial expressions appears important for realistic patient counseling and appropriate technique selection. Accordingly, this study should be interpreted as a descriptive account of clinical experience in carefully selected early aging patients, rather than a comparative or outcome-driven evaluation of the efficacy of the technique.

This study has several limitations. First, its retrospective design limits the ability to apply standardized satisfaction or longevity metrics across the entire cohort. Objective outcome instruments, such as the FACE-Q or GAIS, were not available during the early years of this series and therefore could not be consistently applied. Moreover, follow-up visits were not scheduled at uniform intervals, and no survival-type or time-to-event analysis was performed, so durability can only be described qualitatively rather than quantified with formal time-to-recurrence metrics. In addition, patient satisfaction was captured using a pragmatic, non-validated 5-point Likert scale recorded during routine clinical encounters rather than a formal psychometric instrument, and these retrospective ratings are vulnerable to recall and observer bias. The absence of validated outcome measures and objective quantitative assessments further limits the ability to draw definitive conclusions regarding efficacy. Second, photographic documentation, especially in older cases, is not fully standardized with respect to lighting, positioning, or follow-up timing. In addition, habitual eyebrow grooming or plucking is very common among patients seeking facial rejuvenation and may have a minor influence on the apparent eyebrow contour, which cannot be fully controlled in a retrospective photographic analysis. Given the 30-year time span, identical photographic conditions could not always be ensured, although images were selected to provide comparable views within each patient. Third, overall aesthetic improvements cannot be attributed solely to the suture suspension technique. Adjunctive procedures, particularly micro-fat grafting and laser resurfacing, likely contributed to changes in volume and skin quality and should be considered integral components of the multimodal rejuvenation protocol used in many patients. Finally, the absence of a control or comparison group limits the strength of conclusions regarding efficacy relative to other modalities, and the overlapping use of different interventions prevented robust subgroup analyses, including a separate analysis restricted to patients treated with suture suspension alone. Future prospective studies with standardized outcome measures and defined treatment arms would be valuable to clarify the independent contribution of each component.

## Conclusion

Prolene suture suspension, used as part of a minimally invasive facial rejuvenation protocol, appears to represent a safe and well-tolerated option within a broader multimodal facial rejuvenation strategy. In this 30-year single-surgeon series of 4400 procedures, patient satisfaction was high and the rate of true complications was low, with most adverse events managed successfully under local anesthesia. The technique integrates readily with adjunctive procedures such as micro-fat grafting, micro-liposuction, and fractional laser resurfacing, which likely contribute to the overall rejuvenation effect. These findings should be interpreted in light of the retrospective design, variable follow-up, multimodal treatment patterns, and the absence of a control group, and future prospective controlled studies are needed to more rigorously characterize long-term efficacy and durability of this multimodal approach and to clarify the independent contribution of the suture suspension component.

## Supplementary Information

Below is the link to the electronic supplementary material.

Supplementary file 1 (MP4 91386 KB)
